# Contribution of the BioFire® FilmArray® Pneumonia Panel Plus to the Molecular Characterization of Patients With Lower Respiratory Tract Infections in Northern Morocco

**DOI:** 10.7759/cureus.104524

**Published:** 2026-03-02

**Authors:** Reda Amrani Souhli, El Mehdi El Ghorba, Mohammed Jalal Kouchene, Moussab Arbai, Kawtar El Harrak, Nouhaila Chahid, Hicham Tahoune, Karima Rissoul

**Affiliations:** 1 Microbiology Laboratory, Mohammed VI University Hospital Center, Tangier, MAR; 2 Microbiology, Faculty of Medicine and Pharmacy, Abdelmalek Essaadi University, Tangier, MAR

**Keywords:** co-infections, diagnostic test, filmarray pneumonia plus, gram-negative bacteria (gnb), multiplex pcr, northern morocco

## Abstract

Background

Lower respiratory tract infections are a major cause of hospitalization and mortality worldwide. Identifying the specific causative pathogens remains a significant challenge in clinical practice. This study aimed to establish the epidemiological profile of hospitalized patients with suspected lower respiratory tract infections in northern Morocco by analyzing pathogen distribution, co-detections, and antimicrobial resistance genes, with variations according to age and season.

Methods

A retrospective descriptive study was conducted over a two-year period from December 2023 to November 2025 in the Microbiology Laboratory of Mohammed VI University Hospital Center, Tangier, Morocco. In total, 258 respiratory specimens (sputum, bronchoalveolar lavage, and mini-bronchoalveolar lavage) from hospitalized patients with suspected lower respiratory tract infection were analyzed using the BioFire® FilmArray® Pneumonia Panel Plus (BioFire Diagnostics, LLC, Salt Lake City, UT, USA). The panel targets 27 respiratory pathogens and seven resistance genes. Data were analyzed using descriptive statistics and Pearson’s chi-square and Fisher’s exact tests as appropriate, with a p-value < 0.05 considered statistically significant.

Results

The diagnostic yield was high, with at least one pathogen detected in 205 (79.5%) specimens. Co-detections were identified in 139 (52.7%) cases, with the highest frequency observed in children under five years of age and in elderly patients. The microbial landscape was largely dominated by opportunistic Gram-negative bacilli, particularly the *Klebsiella pneumoniae* group, 89 (34.5%), and the *Acinetobacter baumannii* complex, 81 (31.4%), which showed a consistent, non-seasonal distribution. Resistance genes were detected in 133 (51.6%) patients, with a predominance of CTX-M and NDM, detected in 63 (24.4%) and 57 (22.1%) samples, respectively.

Conclusion

This study improved understanding of the distribution of pathogens causing lower respiratory tract infections in hospitalized patients and underscores the utility of multiplex polymerase chain reaction (PCR) for respiratory pathogen identification, providing valuable insights for epidemiological surveillance and diagnosis.

## Introduction

Lower respiratory tract infections (LRTIs) are among the leading causes of morbidity and mortality worldwide, particularly at both extremes of age. Given their high incidence, potentially severe clinical manifestations, and the substantial burden they impose on healthcare infrastructure, LRTIs constitute a critical public health challenge [1,2]. Establishing a precise etiological diagnosis is frequently complicated by the vast diversity of causative pathogens and the similarity of clinical presentations.

From a microbiological perspective, LRTIs may be caused by a broad range of infectious agents, including bacteria and viruses. While traditional diagnostic approaches - most notably culture-based methods - remain staples of clinical practice, they are hampered by significant limitations, particularly reduced sensitivity in patients who have initiated antibiotic therapy [3,4]. Consequently, molecular diagnostics utilizing syndromic multiplex polymerase chain reaction (PCR) assays have emerged as transformative tools. By enabling the rapid, simultaneous detection of multiple respiratory pathogens, these assays facilitate more timely and informed clinical decision-making [5,6].

Among these tools, the BioFire® FilmArray® Pneumonia Panel Plus (FA-PP+) (BioFire Diagnostics, LLC, Salt Lake City, UT, USA) is one of the most extensively studied molecular panels for the diagnosis of LRTIs. This fully automated multiplex PCR assay allows the rapid detection of a wide range of respiratory pathogens, as well as selected antimicrobial resistance genes [7]. In this study, our primary objective was to establish the epidemiological profile of hospitalized patients with suspected LRTIs in northern Morocco using the FA-PP+, including pathogen distribution, co-detection patterns, and antimicrobial resistance gene targets. Secondary objectives included analyzing age- and season-related variations in detection rates.

## Materials and methods

Study design

This was a retrospective descriptive study conducted over a period of two years, from December 2023 to November 2025, in the Microbiology Laboratory of Mohammed VI University Hospital Center, Tangier, Morocco.

Study population and types of specimens

This study included 258 respiratory specimens collected from hospitalized patients at Mohammed VI University Hospital Center, Tangier, with clinical suspicion of LRTI for whom multiplex PCR testing using the FA-PP+ was requested. Inclusion criteria were based on respiratory symptoms such as cough, persistent fever, and chest pain, as well as severity indicators including respiratory distress, tachypnoea, oxygen desaturation (≤90%), abnormal lung auscultation, and suggestive radiological findings. Specimens included sputum and invasive respiratory samples, notably bronchoalveolar lavage (BAL) and mini-BAL. All samples were collected and transported according to standard procedures and processed in accordance with the manufacturer’s instructions.

Data collection and variables studied

Data collected from the laboratory database included demographic, characteristics (age, sex), the requesting clinical department, type of specimen, detected pathogen, the presence of co-infections, sampling period, as well as available clinical and biological parameters. Pathogen detection was performed using multiplex PCR with the FA-PP+, in accordance with the manufacturer’s instructions [8]. After sample homogenization, a predefined volume was loaded into a single-use cartridge, enabling a fully automated analytical process.

The FA-PP+ allows the simultaneous detection of 27 respiratory pathogens and seven resistance genes. The targeted bacterial pathogens include the *Acinetobacter calcoaceticus-baumannii *complex, *Enterobacter cloacae *complex, *Escherichia coli*, *Haemophilus influenzae*, *Klebsiella aerogenes*, *Klebsiella oxytoca*, the *Klebsiella pneumoniae *group, *Moraxella catarrhalis*, *Proteus *spp*.*, *Pseudomonas aeruginosa*, *Serratia marcescens*, *Staphylococcus aureus*, *Streptococcus agalactiae*, *Streptococcus pneumoniae*, and *Streptococcus pyogenes*. Atypical bacteria detected qualitatively include *Chlamydia pneumoniae*, *Legionella pneumophila*, and *Mycoplasma pneumoniae*. The panel also detects respiratory viruses such as adenovirus, coronaviruses, human metapneumovirus, human rhinovirus/enterovirus (HRV/EV), influenza A and B viruses, parainfluenza viruses, and respiratory syncytial virus (RSV). In addition, the resistance genes screened include carbapenemases (IMP, KPC, NDM, OXA-48-like, and VIM), extended-spectrum β-lactamases of the CTX-M type, as well as methicillin resistance determinants (mecA/C and MREJ) associated with methicillin-resistant *S. aureus*.

Statistical analysis

Data were entered into Microsoft Excel (Microsoft Corp., Redmond, WA, USA) and analyzed using IBM SPSS Statistics for Windows, Version 26 (Released 2018; IBM Corp., Armonk, New York, United States). Categorical variables were presented as frequencies and percentages, whereas quantitative variables were reported as means, medians, and interquartile ranges. Comparison of proportions was performed using Pearson’s chi-square (χ^2^) test or Fisher’s exact test when the assumptions for the chi-square test were not met (expected cell count <5). A p-value < 0.05 was considered statistically significant. The choice between tests was based on expected cell counts and was not intended for comparative methodological evaluation.

Ethical considerations

This retrospective study was conducted on-site with the approval of the head of the department. It was based exclusively on anonymized laboratory data and involved no direct patient contact or intervention. Patient anonymity was ensured, and all data were handled confidentially in accordance with applicable ethical standards and institutional regulations.

## Results

Demographic and clinical characteristics of patients

During the study period, 258 samples from hospitalized patients were analyzed. The mean age of patients was 25.3 ± 23.6 years (range: 1 day to 94 years), with a slight male predominance, accounting for 140 (54.3%); the male-to-female sex ratio was 1.19. Samples were mainly obtained from the pediatric emergency department (58, 22.5%), followed by the neonatal intensive care unit (ICU) (51, 19.7%), the internal medicine department (44, 17.0%), and the adult ICU (32, 12.4%).

Respiratory distress was the main indication for performing multiplex PCR, present in 130 of 258 patients (53.9%). The frequency of this indication varied significantly according to age (p < 0.05), with a high prevalence at both extremes of age, namely patients aged 60 years and older, 26 of 38 (68.4%), and children under five years of age, 75 of 130 (57.7%). Table 1 summarizes the demographic and clinical characteristics of the study population.

**Table 1:** Demographic and clinical characteristics of patients included in the study

Characteristics		Patients (N = 258)	Percentage (%)
Gender	Male	140	54.3
	Female	118	45.7
Age group	<5 years	130	50.4
	5-17 years	10	3.9
	18-59 years	80	31.0
	≥60 years	38	14.7
Symptoms	Cough	72	27.9
	Fever	70	27.1
	Dyspnea	139	53.9
	Polypnea	85	32.9
	Chills	22	8.5
	Anorexia	18	7.0
	Asthenia	22	8.5
Desaturation ≤ 90%	-	51	19.8
Departments	Pediatric Emergency	58	22.5
	Neonatal ICU	51	19.7
	Internal Medicine	44	17.0
	Adult ICU	32	12.4
	Pulmonology	27	10.5
	Pediatric	27	10.5
	Adult Emergency	6	2.3
	Nephrology	8	3.1
	Hematology	3	1.2
	Dermatology	1	0.4
	Thoracic Surgery	1	0.4
Season of admission	Autumn	86	33.3
	Winter	44	17.1
	Spring	74	28.7
	Summer	54	20.9

Positivity rate of respiratory specimens

Of all specimens, 205 (79.5%) were positive. The positivity rate was highest among children under five years of age, with 109 (83.8%), and showed a progressive decrease across age groups, without a statistically significant difference (p = 0.312), suggesting a high and relatively homogeneous circulation of pathogens across the entire study population (Table 2).

**Table 2:** Distribution of positive samples and co-infections by age group (n = 258)

Age group	Total number of specimens	Number of negative specimens, n (%)	Number of positive specimens, n (%)	Number of co-infections, n (%)
<5 years	130	21 (16.2)	109 (83.8)	77 (59.2)
5-17 years	10	2 (20.0)	8 (80.0)	3 (30.0)
18-59 years	80	20 (25.0)	60 (75.0)	37 (46.3)
≥60 years	38	10 (26.3)	28 (73.7)	19 (50.0)
Total	258	53 (20.5)	205 (79.5)	136 (52.7)

Distribution of respiratory pathogens

Among positive samples, co-infections were identified in 136 (52.7%) cases, with a total of 488 pathogens detected. Bacteria accounted for the majority of detections, with 376 (77.0%), dominated by the *K. pneumoniae *group with 89 (23.7%), followed by *A. baumannii *complex with 81 (21.5%), *H. influenzae* with 36 (9.6%), and *P. aeruginosa* with 35 (9.3%). Viruses represented 112 (23.0%) of detections, with a predominance of HRV/EV, accounting for 75 (67.0%), and RSV, accounting for 11 (9.8%).

The prevalence of *A. baumannii *complex and the *K. pneumoniae *group was significantly higher among children under five years, reaching 67 (51.5%) and 64 (49.2%), respectively, compared with other age groups (p < 0.001). Regarding viral pathogens, HRV/EV was the most frequently detected virus, with 75 (29.1%), and affected age groups homogeneously (p = 0.768). This was followed by RSV, with detection rates of nine (6.9%) in children under five years and one (10.0%) in the 5-17-year age group (Table 3).

**Table 3:** Distribution of respiratory pathogens and resistance genes by age group * P-values obtained using Fisher’s exact test due to small expected cell counts NA: not applicable; MERS-CoV: Middle East respiratory syndrome coronavirus; RSV: respiratory syncytial virus

Detected pathogens	<5 years (n = 130), n (%)	5-17 years (n = 10), n (%)	18-59 years (n = 80), n (%)	≥60 years (n = 38), n (%)	Total (N = 258), n (%)	Chi-square (χ^2^) value	p-value
Bacteria							
*Acinetobacter baumannii *complex	67 (51.5)	0 (0)	8 (10.0)	6 (15.8)	81 (31.4)	50.4	<0.001
*Enterobacter cloacae* complex	19 (14.6)	0 (0)	9 (11.3)	2 (5.3)	30 (11.6)	3.95	0.266
Escherichia coli	4 (3.1)	0 (0)	6 (7.5)	5 (13.2)	15 (5.8)	6.55	0.088
Haemophilus influenzae	11 (8.5)	3 (30.0)	15 (18.8)	7 (18.4)	36 (14.0)	7.57	0.056
Klebsiella aerogenes	2 (1.5)	0 (0)	0 (0)	0 (0)	2 (0.8)	-	0.686*
Klebsiella oxytoca	6 (4.6)	0 (0)	0 (0)	1 (2.6)	7 (2.7)	-	0.222*
*Klebsiella pneumoniae *group	64 (49.2)	1 (10.0)	17 (21.3)	7 (18.4)	89 (34.5)	25.7	<0.001
Moraxella catarrhalis	4 (3.1)	1 (10.0)	4 (5.0)	2 (5.3)	11 (4.3)	-	0.398*
*Proteus *spp.	1 (0.8)	0 (0)	1 (1.3)	1 (2.6)	3 (1.2)	-	0.618*
Pseudomonas aeruginosa	16 (12.3)	2 (20.0)	12 (15.0)	5 (13.2)	35 (13.6)	0.67	0.879
Serratia marcescens	2 (1.5)	0 (0)	3 (3.8)	1 (2.6)	6 (2.3)	-	0.630*
Staphylococcus aureus	8 (6.2)	1 (10.0)	14 (17.5)	5 (13.2)	28 (10.9)	6.84	0.077
Streptococcus agalactiae	5 (3.8)	0 (0)	3 (3.8)	2 (5.3)	10 (3.9)	-	0.881*
Streptococcus pneumoniae	8 (6.2)	0 (0)	9 (11.3)	4 (10.5)	21 (8.1)	2.90	0.408
Streptococcus pyogenes	0 (0)	0 (0)	0 (0)	0 (0)	0 (0)	NA	NA
Atypical bacteria							
Chlamydia pneumoniae	0 (0)	0 (0)	0 (0)	0 (0)	0 (0)	NA	NA
Legionella pneumophila	0 (0)	0 (0)	0 (0)	1 (2.6)	1 (0.4)	-	0.186*
Mycoplasma pneumoniae	1 (0.8)	0 (0)	0 (0)	0 (0)	1 (0.4)	-	1.000*
Viruses							
Adenovirus	3 (2.3)	0 (0)	1 (1.3)	0 (0)	4 (1.6)	-	1.000*
Coronavirus	4 (3.1)	0 (0)	1 (1.3)	3 (7.9)	8 (3.1)	-	0.249*
Human metapneumovirus	3 (2.3)	0 (0)	0 (0)	1 (2.6)	4 (1.6)	-	0.466*
Rhinovirus/Enterovirus	38 (29.2)	4 (40.0)	24 (30.0)	9 (23.7)	75 (29.1)	1.15	0.765
Influenza A virus	0 (0)	0 (0)	0 (0)	0 (0)	0 (0)	NA	NA
Influenza B virus	1 (0.8)	0 (0)	0 (0)	0 (0)	1 (0.4)	-	1.000*
MERS-CoV	0 (0)	0 (0)	0 (0)	0 (0)	0 (0)	NA	NA
Parainfluenza virus	4 (3.1)	1 (10.0)	3 (3.8)	1 (2.6)	9 (3.5)	-	0.594*
RSV	9 (6.9)	1 (10.0)	0 (0)	1 (2.6)	11 (4.3)	-	0.027*
Resistance genes							
MECA/C MREJ	0 (0)	0 (0)	1 (1.3)	0 (0)	1 (0.4)	-	0.496*
IMP	0 (0)	0 (0)	0 (0)	0 (0)	0 (0)	NA	NA
KPC	0 (0)	0 (0)	0 (0)	0 (0)	0 (0)	NA	NA
NDM	50 (38.5)	1 (10.0)	6 (7.5)	0 (0)	57 (22.1)	41.8	<0.001
OXA 48	3 (2.3)	0 (0)	1 (1.3)	0 (0)	4 (1.6)	-	1.000*
VIM	6 (4.6)	0 (0)	1 (1.3)	1 (2.6)	8 (3.1)	-	0.588*
CTX-M	50 (38.5)	1 (10.0)	11 (13.8)	1 (2.6)	63 (24.4)	29.7	<0.001

Co-infections

As shown in Table 2, co-infections were more frequent in children under five years of age (77, 59.2%) and in patients aged 60 years and older (19, 50.0%). However, this age-related variation did not show any statistical significance (p = 0.147).

In children under five years, a marked co-detection was observed between *A. baumannii *complex and the* K. pneumoniae *group (46, 35.4%; p < 0.001), compared with other age groups, followed by the association between HRV/EV and *K. pneumoniae *group (19, 14.6%; p = 0.083). In contrast, in adults aged 18-59 years, the most common co-detection associations were HRV/EV - *H. influenzae *(7, 8.8%) (Table 4).

**Table 4:** Distribution of major pathogen co-detections across age groups * P-values obtained using Fisher’s exact test due to small expected cell counts. HRV/EV: human rhinovirus/enterovirus; RSV: respiratory syncytial virus

Type of co-detection	<5 years (n = 130), n (%)	5-17 years (n = 10), n (%)	18-59 years (n = 80), n (%)	≥60 years (n = 38), n (%)	Total, n (%)	Chi-square (χ^2^) value	P-value
Bacteria-Bacteria							
*Acinetobacter baumannii *complex*-Klebsiella pneumoniae *group	46 (35.4)	0 (0.0)	6 (7.5)	2 (5.3)	54 (20.9)	33.4	<0.001
*Enterobacter cloacae *complex*-K. pneumoniae *group	13 (13.1)	0 (0.0)	6 (3.8)	2 (2.6)	21 (8.1)	1.95	0.582
*A. baumannii *complex*-E. cloacae *complex	14 (10.8)	0 (0.0)	4 (5.0)	2 (5.3)	20 (7.8)	3.67	0.299
*K. pneumoniae *group*-Pseudomonas aeruginosa*	12 (9.2)	0 (0.0)	2 (2.5)	0 (0.0)	14 (5.4)	7.76	0.051
*A. baumannii *complex*-P. aeruginosa*	10 (7.7)	0 (0.0)	2 (2.5)	1 (2.6)	13 (5.0)	3.98	0.263
*Escherichia coli-K. pneumoniae *group	4 (3.1)	0 (0.0)	2 (2.5)	4 (10.5)	10 (3.9)	-	0.177*
Haemophilus influenzae-Staphylococcus aureus	4 (2.3)	0 (0.0)	5 (3.8)	1 (10.5)	10 (3.9)	-	0.699*
H. influenzae-S. pneumoniae	6 (2.3)	0 (0.0)	2 (3.8)	1 (7.9)	9 (3.5)	-	0.926*
S. aureus-Streptococcus pneumoniae	3 (2.3)	0 (0.0)	3 (3.8)	1 (2.6)	7 (2.7)	-	0.902*
*E. cloacae *complex*-P. aeruginosa*	5 (1.5)	0 (0.0)	2 (5.0)	0 (2.6)	7 (2.7)	-	0.755*
Virus-Bacteria							
HRV/EV-*K. pneumoniae* group	19 (14.6)	1 (10.0)	3 (3.8)	3 (7.9)	26 (10.1)	6.69	0.083
HRV/EV-*H. influenzae*	7 (5.4)	2 (20.0)	7 (8.8)	3 (7.9)	19 (7.4)	3.33	0.344
HRV/EV-*A. baumannii *complex	17 (10.0)	0 (0.0)	0 (6.3)	1 (0.0)	18 (7.0)	15.3	0.002
HRV/EV-*P. aeruginosa*	6 (4.6)	1 (10.0)	6 (7.5)	0 (0.0)	13 (5.0)	3.59	0.309
HRV/EV-*E. cloacae* complex	7 (3.8)	0 (10.0)	1 (3.8)	1 (0.0)	9 (3.5)	-	0.454*
HRV/EV-*S. aureus*	5 (2.3)	1 (10.0)	1 (2.5)	2(7.9)	9 (3.5)	2.85	0.415
HRV/EV-*S. pneumoniae*	4 (2.3)	0 (0.0)	4 (3.8)	0 (5.3)	8 (3.1)	-	0.547*
HRV/EV-*Moraxella catarrhalis*	2 (1.5)	0 (0.0)	3 (3.8)	2 (5.3)	7 (2.7)	-	0.447*
RSV-*K. pneumoniae* group	6 (4.6)	0 (0.0)	0 (0.0)	0 (0.0)	6 (2.3)	-	0.155*
RSV-*H. influenzae*	4 (0.8)	1 (0.0)	0 (0.0)	0 (10.5)	5 (1.9)	-	0.098*
Virus-Virus							
Adenovirus-HRV/EV	3 (0.8)	0 (0.0)	1 (2.5)	0 (2.6)	4 (1.6)	-	1.000*
HRV/EV-RSV	3 (1.5)	1 (0.0)	0 (1.3)	0 (2.6)	4 (1.6)	-	0.114*
HRV/EV-Parainfluenza virus	3 (2.3)	0 (0.0)	1 (1.3)	0 (0.0)	4 (1.6)	-	1.000*

Resistance genes

Resistance genes were detected in 133 (51.6 %) of patients, with a predominance of CTX-M genes (63, 24.4%), followed by NDM gene (57, 22.1%). The distribution of CTX-M and NDM showed a significant association with age (<0.001). They were frequent in patients under five years, with a prevalence of 50/130 (38.5%) each (Table 3).

Seasonality

The most detected pathogens, such as *K. pneumoniae *group, *A. baumannii *complex, and HRV/EV, circulated all over the period of the study with no statistical significance(p > 0.05). In contrast, RSV showed a marked winter peak of 5/44 (11.4%), extending into spring 6/74 (8.1%; p = 0.002), while *S. aureus* (p = 0.009) and *M. catarrhalis* (p = 0.025) reached their respective prevalence peak of 10/44 (22.7%) and 6/44 (13.6%) during the same season.

**Figure 1:**
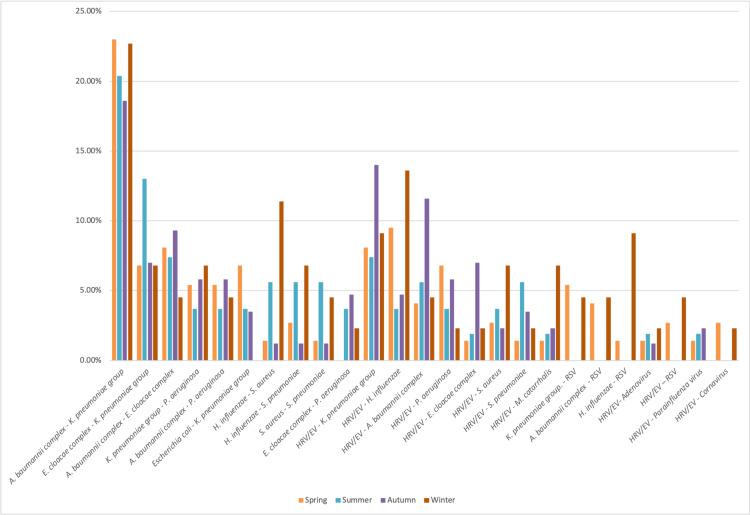
Seasonal distribution of the main pathogen co-detections across the four seasons HRV/EV: human rhinovirus/enterovirus; RSV: respiratory syncytial virus

The majority of observed associations involved bacterial pathogens and showed a homogeneous distribution across four seasons, without significant seasonal variation. Associations involving both viral and bacterial pathogens were mainly driven by HRV/EV, especially in combination with the *K. pneumoniae *group and *H. influenzae*. In contrast, the association of *H. influenzae* and RSV was more frequent during the winter season (4, 9.1%). The viral combination was uncommon and scattered throughout the year (Figure 1).

## Discussion

In our study, the overall positivity rate was high (79.5%). This result is similar to findings from several reported studies using the FA-PP+, which described high detection rates of respiratory pathogens in hospitalized settings [7,9-13]. Ginocchio et al. and Webber et al. found the detection of at least one bacterial or viral pathogen in 70.57% and 58.5% of analyzed samples, respectively [10,12].

From the positive samples, bacteria were the most commonly detected pathogens (77.2%), with predominance of the *K. pneumoniae *group and *A. baumannii *complex. This pattern is typical of hospital-acquired LRTIs, especially in ICUs, where multidrug-resistant Gram-negative bacteria are often involved [14]. Our results are consistent with several hospital-based studies, especially in resource-limited settings, in which *K. pneumoniae* and *A. baumannii* are among the leading causes of severe LRTIs [13,14], likely reflecting healthcare-associated infections.

A notable epidemiological finding of our study is the high concentration of *A. baumannii *complex (51.5%) and *K. pneumoniae *group (49.2%) among children under five years of age (p < 0.001), suggesting either early nosocomial transmission or increased vulnerability of this population.

Viruses accounted for 23% of detected agents, with a predominance of HRV/EV, followed by RSV. Rhinovirus is recognized as one of the most frequently detected respiratory pathogens in hospitalized children [15]. RSV was mainly identified in children under five years of age, confirming its major role in severe LRTIs in infants [16,17].

The rate of co-detected pathogens was high (52.7%). Compared with other studies, this rate is concordant and may be due to the multiple targets that can be tested in a single assay [9,10,12,13]. Co-detections were more frequent in children under five years and in older patients. Rapid detection of these co-infections using syndromic panels provides a clear overview of the pathogens involved and may help to improve and adjust the adaptation of therapeutic management [6,18]. In children under five years, co-detections involving the *A. baumannii *complex and *K. pneumoniae *group were most commonly observed, likely reflecting increased hospital exposure and immature immune defenses, as reported in pediatric studies of severe LRTIs [1,19]. The frequent detection of HRV/EV supported the well-established role of respiratory viruses in predisposing to secondary bacterial infection through epithelial damage and immune modulation [20]. Among adults aged 18-59 years, co-detections mainly involved opportunistic Gram-negative bacteria such as *K. pneumoniae* and *P. aeruginosa*, often found alongside respiratory viruses. This pattern likely reflects a combination of community - and hospital - acquired infections influenced by comorbid conditions, previous antibiotic exposure, and invasive respiratory procedures, as described in earlier studies of adult pneumonia [21,22]. In patients aged 60 years and older, the co-detections involving RSV highlighted the increased vulnerability of elderly individuals to viral-bacterial interactions. RSV infection in older adults has been increasingly recognized as a trigger for bacterial superinfection due to immunosenescence and impaired mucociliary clearance [23,24]. Overall, these findings confirm a clear age-dependent distribution of bacterial and bacterial-viral co-detections and underline the importance of age-adapted diagnostic and therapeutic strategies in LRTIs.

The detection of resistance genes in more than half of patients (51.6%) is a matter of concern. The predominance of CTX-M (24.4%) and NDM (22.1%) genes observed in our study is consistent with international data describing the widespread dissemination of extended-spectrum β-lactamases and carbapenemases among Enterobacterales and *A. baumannii*. Several studies have highlighted the high burden of multidrug-resistant bacteria in African countries and other resource-limited regions [25,26]. The integration of resistance gene detection into the FA-PP+, therefore, represents a valuable complementary tool for early guidance and adjustment of antibiotic therapy, as well as for strengthening antimicrobial stewardship programs [18,27].

Finally, analysis of seasonal distribution showed relatively stable circulation of the main bacterial pathogens throughout the year, suggesting an endemic and predominantly nosocomial pattern [13,14]. In contrast, RSV exhibited marked winter predominance, in line with global epidemiological data [16,17]. Seasonal variations observed for certain pathogens may reflect complex interactions between viral infections, bacterial colonization, and environmental factors, as described by Bosch et al. [20].

The stable, non-seasonal nature of the associations we observed between the *A. baumannii *complex and the *K. pneumoniae *group points toward an endemic circulation within the hospital environment. This is a common feature of Gram-negative bacteria linked to severe or healthcare-associated LRTIs [28]. Conversely, the fact that the* H. influenzae*-RSV association peaked during the winter months aligns perfectly with the known seasonal surge of RSV and its well-documented role in facilitating secondary bacterial complications [29].

Limitations

The retrospective, single-center design of this study and the high proportion of ICU patients may limit the generalizability of the findings, and likely contributed to the high prevalence of nosocomial and multidrug-resistant bacteria. Furthermore, the FA-PP+ is based on molecular detection of nucleic acid and does not allow a clear distinction between active infection and colonization. Finally, the absence of systematic comparison with culture or other diagnostic methods limits the assessment of diagnostic concordance and the direct impact of the test on antimicrobial therapy and clinical outcomes.

## Conclusions

This study demonstrated that LRTIs in hospitalized patients may be caused by a wide spectrum of pathogens, predominantly bacteria. The high rate of co-infections and the frequent detection of antimicrobial resistance genes strongly suggest a nosocomial origin of these infections. In this context, the FA-PP+ proved useful in improving the microbiological characterization of LRTIs in a hospital setting. Further studies conducted in low- and middle-income countries are needed to better assess its impact on antibiotic therapy optimization and overall patient management.
